# Hepatic Transcriptome Reveals Potential Key Genes Contributing to Differential Milk Production

**DOI:** 10.3390/genes15091229

**Published:** 2024-09-20

**Authors:** Chao Du, A La Teng Zhu La, Shengtao Gao, Wenshuo Gao, Lu Ma, Dengpan Bu, Wenju Zhang

**Affiliations:** 1 College of Animal Science and Technology, Shihezi University, Shihezi 271018, China; duchao105@163.com; 2 State Key Laboratory of Animal Nutrition and Feeding, Institute of Animal Science, Chinese Academy of Agricultural Sciences, Beijing 100193, China; malu.nmg@163.com; 3 Inner Mongolia Key Laboratory of Animal Nutrition and Feed Science, College of Animal Science, Inner Mongolia Agricultural University, Hohhot 010018, China; zhula8336@163.com; 4 College of Life Science and Technology, Inner Mongolia Normal University, Hohhot 010018, China; shengtaogao@163.com (S.G.); wenshuogao0711@163.com (W.G.); 5 Key Laboratory of Biodiversity Conservation and Sustainable Utilization in Mongolian Plateau for College and University of Inner Mongolia Autonomous Region, Hohhot 010018, China

**Keywords:** hepatic transcriptome, milk production, lactation initiation, WGCNA, breeding

## Abstract

Background: Despite the widespread adoption of TMR or PMR and the formulas designed to sufficiently cover the cows’ requirements, individual dairy cows’ milk production varies significantly. The liver is one of the most important organs in cow lactation metabolism and plays an essential role in the initiation of lactation. Objectives: This study aimed to investigate the potential key genes in the liver contributing to the different milk production. Methods: We enrolled 64 cows and assigned them to high or low milk yield (MY) groups according to their first 3 weeks of milk production. We performed RNAseq for 35 liver samples with 18 from prepartum and 17 from postpartum cows. Results: The continuous milk yield observation showed a persistently higher milk yield in high MY cows than low MY cows in the first 3 weeks. High MY cows showed better feed conversion efficiency. We identified 795 differentially expressed genes (DGEs) in the liver of high MY cows compared with low MY cows, with up-regulated genes linked to morphogenesis and development pathways. Weighted gene co-expression network analysis (WGCNA) revealed four gene modules positively correlating with milk yield, and protein and lactose yield (*p* < 0.05). Using the intersected genes between the four gene modules and DEGs, we constructed the linear mixed-effects models and identified six hub genes positively associated and two hub genes negatively associated with milk yield (Coefficients > 0.25, *p* < 0.05). Random forest machine learning model training based on these eight hub genes could efficiently predict the milk yield (*p* < 0.001, R^2^ = 0.946). Interestingly, the expression patterns of these eight hub genes remained remarkably similar before and after parturition. Conclusions: The present study indicated the critical role of liver in milk production. Activated processes involved in morphogenesis and development in liver may contribute to the higher milk production. Eight hub genes identified in this study may provide genetic research materials for dairy cow breeding.

## 1. Introduction

It is generally believed that milk yield is positively correlated with DMI (dry matter intake) [1]. The achievement of optimal milk production often relies on the implementation of appropriate and scientifically-based nutritional consumption and rearing management practices. Nowadays, total mixed ration (TMR) or partial mixed ration (PMR) technology has been widely adopted by nearly all dairy farms, and dietary formulations are meticulously designed to meet the nutritional requirements of cows. To optimize the milking environment for cows and enhance milk yield during each milking session, an increasing number of modern dairy farms have implemented the utilization of automated milking systems [2]. Despite this, significant variability in milk production among individual animals remains evident, even when they are fed identical TMR diets. This variability may partly result from the liver’s pivotal role in regulating numerous physiological processes critical for milk production and overall health. The liver acts as a central hub, responsible for the metabolism of macronutrients, regulation of blood volume, immune system support, endocrine control of growth signaling pathways, and the maintenance of lipid and cholesterol homeostasis, as well as the breakdown of xenobiotic toxic compounds [3,4].

During the periparturient period, a time of significant metabolic stress for dairy cows, the liver’s role becomes even more critical [5]. It is essential for controlling and regulating metabolic adaptation, ensuring the cow can meet the increased energy demands associated with parturition and the onset of lactation [6]. The liver is also indispensable for the metabolism of key substances like gluconeogenesis, lipids, and amino acids, which is not only crucial for the initial stages of lactation but also for sustaining milk synthesis throughout the entire process [7,8]. Considering the liver’s significant metabolic and physiological functions, we speculate that differences in liver function among cows may lead to variations in milk production, although the specific genes responsible are not well understood.

Previous studies using RNA sequencing to analyze the liver transcriptome of peripartum dairy cows primarily focused on comparing changes and adaptations before and after parturition and identifying candidate genes associated with milk production initiation. Ha et al. (2017) compared the liver’s transcriptional characteristics across three stages: 3 weeks before expected calving, 2 weeks after calving, and 3 weeks after calving [9]. Their findings suggest that significant changes in liver function observed in peripartum dairy cows are closely linked to energy metabolism, including fatty acid oxidation and metabolism, cholesterol metabolism, and gluconeogenesis [9]. By comparing the liver transcriptomic characteristics before and after calving, the results revealed that pathways related to lipid and glucose metabolism biosynthesis were more affected and activated post-calving, with PPAR and adipokines playing key roles [5]. Furthermore, a comparative analysis of liver transcriptome sequencing in dairy cows during the dry period, early lactation, and peak lactation phases identified a total of 10 genes as potential influencers of milk yield and the traits of milk protein and fat [10]. These studies primarily compared changes in the liver transcriptome of the same cow before and after parturition, without considering variations in the liver transcriptome due to differences in individual milk production. Additionally, they did not identify the genes that have a potential decisive role in high milk production within the liver.

In the current study, we utilized transcriptomics to deeply explore the liver transcriptomes of dairy cows with similar feeding backgrounds yet exhibiting vastly different milk production. The objective was to identify the key genes within the liver that play a decisive role in milk production. From the perspective of organ-level gene expression, this research offers foundational genetic materials for related molecular breeding studies.

## 2. Materials and Methods

### 2.1. Experimental Animals and Management

This study was conducted in conformity to the Animal Care and Use Committee of the Institute of Animal Science, Chinese Academy of Agricultural Sciences (No. IAS20180115, Beijing, China). To explore the genetic factors in the liver that may underlie variations in milk production, we enrolled a prospective cohort (who had similar conditions initially and were not grouped until 3 weeks after parturition) of 64 pregnant third lactation Holstein dairy cows from a large herd with a Body Condition Score (BCS) between 3.25 and 3.5 (1–5 scale). All of the cows selected had similar expected calving dates. Before calving, all of the cows were fed with close-up cows’ diet, and after calving, the diet was changed to fresh cows’ diet (Supplementary Table S1). The cows’ diet in this study was formulated based on the National Research Council’s Nutrient Requirements of Dairy Cattle (NRC, 2001) [11] to meet the nutrient requirements of a prepartum cow weighing 650 kg with 12.01 kg of DM/d consumed and a postpartum cow weighing 580 kg with 16.7 kg of DM/d consumed and 33 kg/d of milk yielded. All of the cows were fed with a total mixed ration (TMR)-based diet, provided twice per day at 06:00 and 14:00. All experimental cows were housed in a ventilated enclosed barn during the experimental period and were individually fed with refusals recorded each morning for 4 consecutive days per week. After calving, the cows were milked 3 times per day with milk yield manually recorded exactly for each milking. Three weeks after parturition, all 64 cows were classified into high milk yield (High MY) and low milk yield (Low MY) groups based on their average milk production over the postpartum three-week period.

Feed conversion efficiency (FCE) was defined as the ratio of energy-corrected milk (ECM) to kg of dry matter intake (DMI). DMI was calculated by quantifying the amount of feed offered and subtracting the orts remaining. The FCE was computed using the subsequent formula:

(1) $$FCE=\frac{milk\;yield\times (383\times fat\%+242\times protein\%+783.2)/3140\;(kg/d\cdot head)}{DMI\left(kg/d\cdot head\right)}$$

To establish a correlation between milk production and FCE, we conducted a linear regression analysis on the dataset of milk yields and FCE. The calculation of ECM and FCE referred to Chen et al. (2024) and Sjaunja et al. (1991) [12,13]. In this formula, the constants of 383 and 242 represent the standard energy of fat and protein. The constant of 783.2 represents the average energy of lactose energy (4.61%) and citric acid (0.2%). The constant of 3140 represents the average energy content of 1 kg FCM reported in various publications.

### 2.2. Blood Sample Collection and Measurement

Blood samples were collected from individual cows at 07:00 h daily on d 21 relative to calving, as described by Girma et al. [14]. All blood samples were drawn into serum separator tubes, permitted to clot for at least 25 min at 20 °C, and subsequently refrigerated overnight for storage. Serum was obtained by centrifuging the blood at 4 °C and 3000× *g* for 15 min and then kept at −20 °C. Aspartate aminotransferase activity (AST, catalog no. C010-2-1), total bilirubin (TBil, catalog no. C019-1-1), and total anti-oxidative capacity (T-AOC, catalog no. A015-2-1) in the serum were analyzed using commercial kits purchased from Nanjing Jiancheng Bioengineering Institute (Nanjing, Jiangsu, China). All of the measurements were conducted exactly according to the standard procedures listed in the user manuals of each kit. The measurement of AST activity was conducted based on the amount of pyruvate transformed from aspartic acid. ABTS is oxidized into green ABTS^+^ under the action of an appropriate oxidant. The T-AOC was estimated according to the inhibiting effect on the formation of ABTS^+^. The oxidation of TBil in the presence of surfactants and vanadates leads to the formation of biliverbin, and the reduction in absorbance detected at 450nm is directly proportional to the concentration of TBil.

### 2.3. Liver Tissue Sample Collection

Almost 21d before calving, 18 cows were randomly selected from the 64 cows for liver tissue collection. Then 21d after parturition, liver samples were collected again from these cows, with the exception of one cow due to multiple unsuccessful attempts during liver collection. Liver tissue samples from before and after parturition were assigned to the high MY group or low MY group based on their average milk production for the first 3 weeks. Consequently, before parturition, 7 liver samples were classified to the high MY group and 11 liver samples were classified to low MY group. For liver samples collected from after parturition, the high MY group and low MY group included 6 and 11 liver samples, respectively. The specific grouping information of all of the liver samples is listed in Supplementary Table S2.

The biopsy was conducted as described by Bu et al. (2017) [15] and Gao et al. (2021) [5]. Briefly, before administering a local anesthetic, the cows were given a low dose of xylazine (0.05 mg/kg body weight). A volume of 3 to 4 milliliters of lidocaine hydrochloride (2% solution) was administered subcutaneously as a local anesthetic prior to making the incision. A 1.5 cm longitudinal incision was then made between the eleventh and twelfth ribs on the cow’s right side using a sterile scalpel. For the liver biopsy after parturition, the new incision was kept away from the old incision cut before parturition to avoid a second incision impeding the wound healing. Sterile gauze was applied to the incision with pressure, which was continued until no visible signs of bleeding remained. Following this, a liver biopsy was performed, resulting in the acquisition of approximately 0.3 g of tissue. Liver tissue samples were washed with phosphate buffered saline buffer prepared using nuclease-free water, and were quick-frozen in liquid nitrogen and then stored to −80 °C for RNA extraction. The incision in the skin performed for the biopsy procedure was securely approximated using a total of four to five metal (Michel) clips. The incision site was treated with a topical antiseptic, specifically a 10% povidone iodine ointment (Taro Pharmaceuticals, Hawthorne, NY, USA). For the 7 days following the biopsy, the cow’s physiological status was meticulously recorded, including rectal temperature and daily dietary intake. The surgical clips were excised 7 days following the biopsy, after which the cows were returned to their respective barns on the farm to resume their rearing.

### 2.4. Transcriptomics Analysis and Data Processing

Total RNA was extracted from each sample using TRIzol reagent (Invitrogen, Carlsbad, CA, USA). The purity, concentration, and integrity of the RNA were assessed utilizing the Nano-Photometer spectrophotometer (IMPLEN, Westlake Village, CA, USA), the Qubit RNA Assay Kit with the Qubit 2.0 Fluorometer (Life Technologies, Carlsbad, CA, USA), and the RNA Nano 6000 Assay Kit on the Bioanalyzer 2100 System (Agilent Technologies, Santa Clara, CA, USA). The construction and sequencing of the cDNA library were performed by the Novogene Bioinformatics Institute (Beijing, China). The complementary DNA (cDNA) library was constructed following the standard Illumina protocol and sequenced with the HiSeq4000 platform (Illumina, San Diego, CA, USA).

Raw data were processed to remove reads with adapters, poly-N reads, and low-quality reads, resulting in a set of clean reads. Subsequently, FastQC (v. 0.12.1) software was employed to determine the Q20, Q30, and GC content of the clean reads, and to assess the overall quality of the sequencing data [16]. The reference genome of bovine (ftp://ftp.ensembl.org/pub/release-89/fasta/bos_taurus/DNA/, accessed on 8 June 2020) and annotation files (ftp://ftp.ensembl.org/pub/releas-89/gtf/bos_taurus, accessed on 8 June 2020) were downloaded. We used Bowtie2 to construct the index of the reference genome, and aligned paired-end clean reads to the reference genome using HISAT2 [17]. StringTie2 was used to assemble the mapped reads of each sample to get the read-count data of the gene expression matrix [18]. Differentially expressed genes (DEGs) were calculated with the DeSeq2 package in R [19], and the screening threshold was *p*-value < 0.05. The R package “clusterProfiler” was employed for GO/KEGG enrichment analyses (*p* adjust  <  0.05) on the DEGs [20].

### 2.5. Construction of Gene Co-Expression Network

To demonstrate the dynamics of genes identified in cows in low and high MY groups, a co-expression module was constructed using the Weighted Gene Co-expression Network Analysis (WGCNA) package of R [21]. The data samples were grouped into clusters to detect outliers. We computed pairwise correlation coefficients between genes and built a weighted adjacency matrix by applying a soft-thresholding power (β). We then utilized hierarchical clustering to create the clustering tree structure for the Topological Overlap Matrix (TOM). The association between gene traits and key modules was measured using gene significance and module membership metrics. Genes with strong correlations to milk-related traits within the most significant modules were selected for subsequent analysis.

### 2.6. Identification and Functional Enrichment Analysis of Intersection Genes between WGCNA Modules and DEGs

The Venn package was utilized to identify the intersecting DEGs and the candidate hub genes from the WGCNA. The R package “clusterProfiler” was employed for GO/KEGG enrichment analyses (*p* adjust  < 0.05) on the intersection genes [20].

### 2.7. Screening the Hub Genes Highly Associated with Milk Yield Using Linear Mixed-Effects Models

Linear mixed-effects models were employed to relate the intersected genes to milk yield using the following formula:

(2) Milk yield~β1(WGCNA∩DEGs)

We used an analysis of variance to determine the statistical significance of the fixed effects in our model, specifically the relationship between WGCNA ∩ DEGs and milk yield. Milk yield-associated hub genes were defined as those with coefficients >|0.3| and *p* < 0.05.

### 2.8. Random Forest Machine Learning Model Validated the Importance of Hub Genes for Milk Production

To further verify the determining role of the hub genes on the variants of milk production, we conducted a 10-fold validated Random Forest machine learning model based on the 8 hub genes using the R package randomForest [22]. Then we predicted the actual milk yield of each cow based on the expression profiles of these 8 hub genes using this model, and evaluated the accuracy of prediction by calculating the association between the predicted milk yield and actual milk yield using liner regression analysis.

### 2.9. Statistical Analysis

The statistical software R version 4.3.4 was used to perform the statistical analysis and visualization of all of the data. Specifically, the linear regression analysis in this study was conducted using the lm function in the R package of stats with the parameters set as default. For the features of FCE, AST, TBil, and TAOC, we first conducted the descriptive statistical analysis and calculated the minimum, first quartile (Q1), median (Q2), third quartile (Q3), and maximum values of each feature. IQR (Q3–Q1) was used to calculate the upper and lower bounds, and data points outside the [Q1−1.5IQR, Q3 + 1.5IQR] range were considered as outliers. Then, these outliers were removed from the following analysis. The differential analysis was performed using lmer function in the R package of lme4 (v. 1.1–35.5) with group and week as fixed effects, cow as a random effect, and *p* < 0.05 indicating statistical significance.

## 3. Results

### 3.1. Milk Yield Positively Correlates with Feed Conversion Efficiency

**Figure 1:**
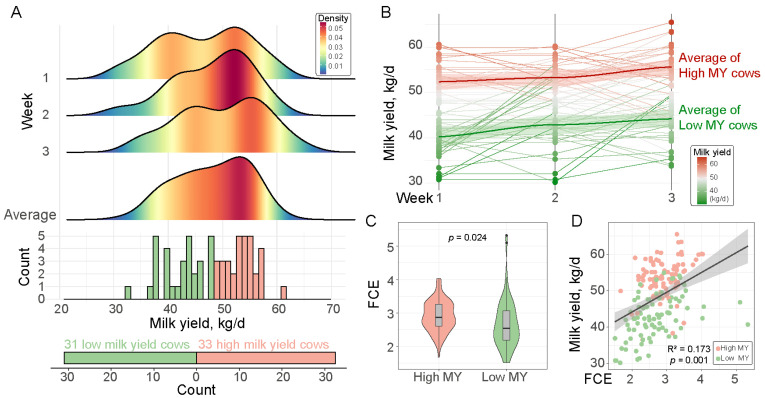
The high MY cows have higher feed conversion efficiency. (**A**) Schematic diagram of milk yield groupings. (**B**) Milk yield during the first three weeks in the high and low MY groups. (**C**) Comparison of the FCE between the high and low MY groups. (**D**) Regression analysis of milk yield and FCE.

In this study, we categorized 64 dairy cows into two groups based on their milk production over the first three weeks: a high milk yield group (High MY, *n* = 33) and a low milk yield group (Low MY, *n* = 31; Figure 1A). The statistical analysis of the milk production data revealed a consistent trend during the first three weeks, with the high MY group exhibiting significantly higher milk production compared to the low MY group (Figure 1A,B). Further, the high MY group had an average milk yield that was 11.14 kg/d higher than the low MY group (Figure 1B). To demonstrate that high MY cows exhibit a higher FCE, we compared the FCE between the high and low MY groups. The results indicated that the High MY group had a significantly greater FCE compared to the low MY group (*p* < 0.05, Figure 1C). Interestingly, we also found a significant association between milk yield and FCE (*p* = 0.001, R^2^ = 0.173, Figure 1D). These findings suggested that cows’ high MY partially contributed to higher FCE.

### 3.2. Liver Health Status and Cows’ Milk Production

**Figure 2:**
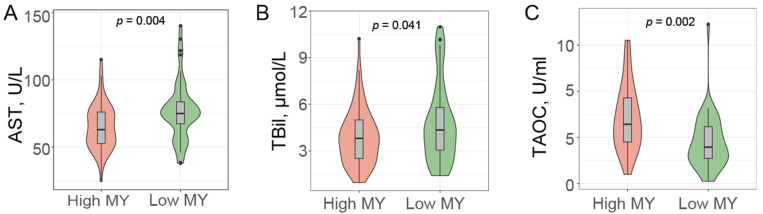
The liver functionality in cows exhibiting high milk yield is elevated. (**A**) AST. (**B**) TBil. (**C**) TAOC.

To demonstrate the potential influence of liver function on the milk production of dairy cows, we measured biomarkers of liver function [23], such as AST and TBil, as well as the TAOC capacity in blood. Compared with the low MY cows, the high MY cows exhibited significantly lower AST and TBil levels (*p* < 0.05, Figure 2A,B) and a significant increase in serum TAOC activity (*p* < 0.05, Figure 2C), implying a higher hepatic health status in high MY cows.

**Figure 3:**
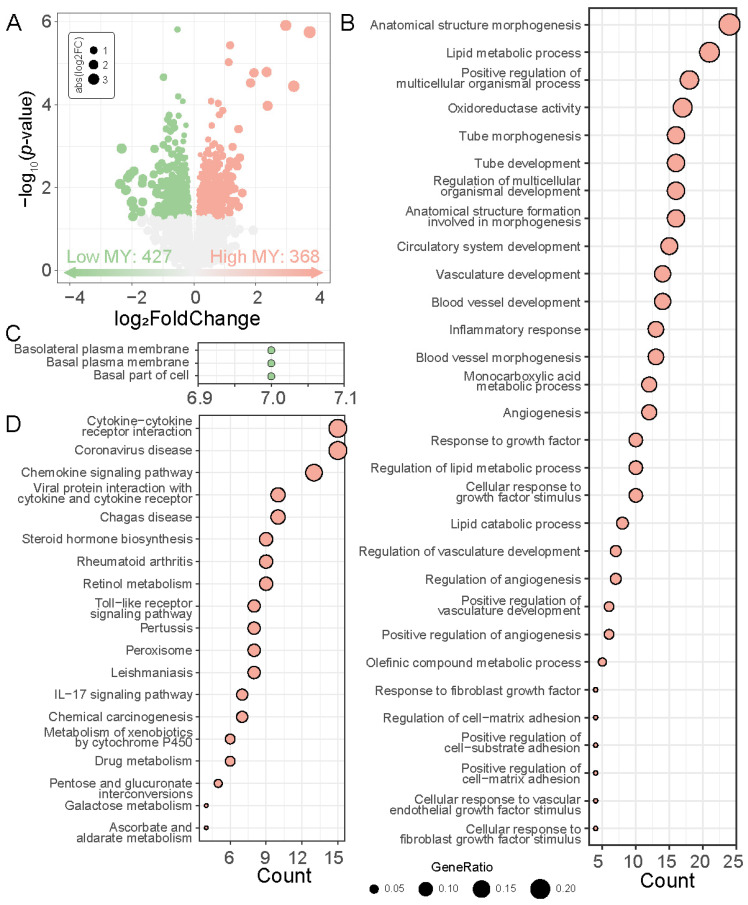
Liver tissue transcriptomic profiles of the low and high MY cows post-calving. (**A**) Volcano plot for the differentially expressed genes (DEGs) of liver tissue of low MY cows compared with high MY cows. Significantly up-regulated and down-regulated DEGs are represented as ‘red’ and ‘green’ dots in the volcano plot, respectively. GO enrichment analysis of DEGs between the (**B**) high MY and (**C**) low MY groups. (**D**) KEGG enrichment analysis of DEGs in the high MY group.

Since liver function affects cow milk production, we wondered if there were any changes in potential key pathways and genes within the liver. We then performed transcriptome sequencing on liver tissue from high and low MY cows three weeks post-calving. By comparing the gene expression profiles in the two groups, a total of 795 DEGs were identified (*p* < 0.05, Figure 3A), among which 368 and 427 genes were up- and down-regulated in the high MY cows, respectively. GO functional analysis indicated that up-regulated genes were mainly associated with Anatomical structure morphogenesis, Lipid metabolic process, Positive regulation of multicellular organismal process, Oxidoreductase activity, and Tube morphogenesis (*p* < 0.05, Figure 3B, Supplementary Table S3), while the down-regulated genes were related to Basolateral plasma membrane, Basal plasma membrane, and Basal part of cell (*p* < 0.05, Figure 3C, Supplementary Table S4). KEGG enrichment analysis showed that the up-regulated genes were significantly enriched in 19 KEGG pathways such as Cytokine−cytokine receptor interaction, Coronavirus disease, Chemokine signaling pathway, Viral protein interaction with cytokine and cytokine receptor, and Chagas disease pathways (*p* < 0.05, Figure 3D, Supplementary Table S5). The down-regulated genes could not be successfully enriched in any KEGG pathways.

### 3.3. WGCNA Construction and Key Module Genes Identification

**Figure 4:**
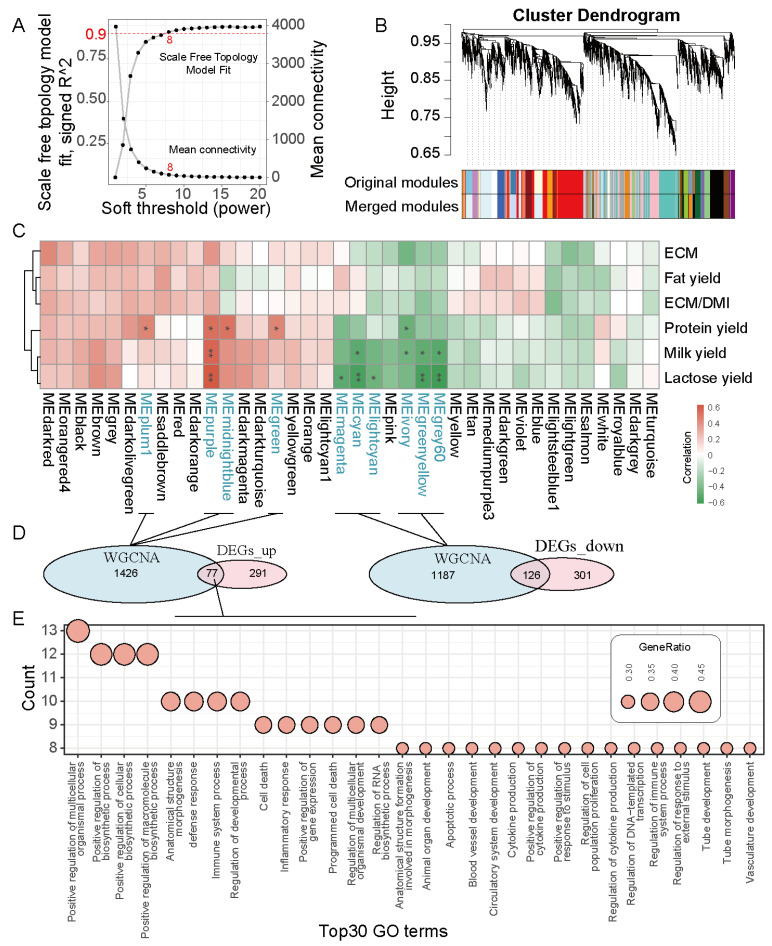
Implementation of WGCNA and identification of key module genes. (**A**) The soft-thresholding power (β) was set to eight, which guaranteed a scale with an R^2^ value of 0.9. (**B**) Dendrogram of co-expression network modules in WGCNA based on dissimilarity metrics. (**C**) Heat map of the correlation between gene co-expression modules and milk production. The red and green boxes represent the gene co-expression modules in the positive and negative directions, respectively. (**D**) Venn diagram for overlapped genes between WGCNA and DEGs. (**E**) GO enrichment analysis was performed on the intersection genes between WGCNA and up-regulated genes. * *p* < 0.05. ** *p* < 0.01.

To further excavate the genes contributing to the variable of milk production, a co-expression network analysis was conducted to identify the gene modules highly associated with milk yield. After calculating the unscaled connectivity index, an average connectivity analysis was performed. At a soft threshold of β = 8, the network reached an unscaled topological connectivity of 0.9, as shown in Figure 4A. By dynamic tree cutting and calculation, 38 gene modules were obtained (Figure 4B). Module–trait relationships illustrate the associations between different gene modules (indicated by colors) and milk production, with each grid representing the correlation between a specific gene module and milk production (Figure 4C). A significant positive correlation was observed between protein yield and four modules: MEplum1, MEpurple, MEmidnightblue, and MEgreen (*p* < 0.05, Figure 4C). For the MEpurple module, the genes assigned to it exhibited a general association with milk yield and lactose yield (*p* < 0.05, Figure 4C). The MEivory module correlated negatively with protein yield (*p* < 0.05, Figure 4C). The MEcyan, MEivory, MEgreenyellow, and MEgrey60 modules demonstrated the most significant negative correlations (*p* < 0.05, Figure 4C). Additionally, the MEmagenta, MEcyan, MElightcyan, MEivory, MEgreenyellow, and MEgrey60 modules had strong negative correlations with lactose yield (*p* < 0.05, Figure 4C). In total, we identified 1,426 genes associated with milk yield, protein yield, and lactose yield, intersected these with the up-regulated DGEs with 77 intersection genes obtained (Figure 4D). Furthermore, we performed functional enrichment analysis on the 77 intersection genes using GO enrichment. The top 30 most enriched GO terms are shown in Figure 4E (Supplementary Table S6). In addition, we also analyzed the intersected genes between WGCNA modules and down-regulated DGEs. Unfortunately, the 126 intersection genes cannot enrich any GO terms.

### 3.4. Hub Genes Further Filtered with Linear Mixed-Effects Model and Verification Using Machine Learning

**Figure 5:**
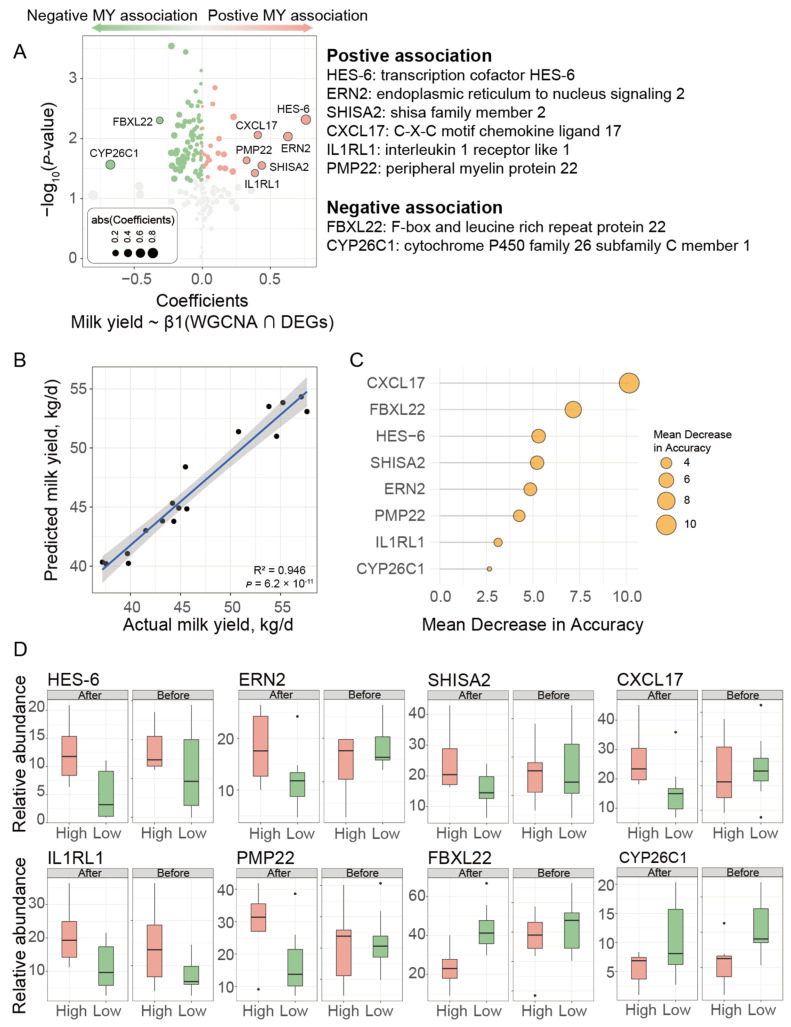
Screening of hub genes. (**A**) The results of linear mixed-effects modelling of the relationship between intersection genes’ abundance and MY. Red and green dots represent intersection genes that are positively and negatively associated with milk yield, respectively. (**B**) Machine learning models for predicting milk yield were fitted. (**C**) The ranking of intersection genes’ importance in the prediction of milk yield. (**D**) The relative abundance of the eight hub genes in the liver tissues of high and low MY cows were compared before and after calving.

We then used linear mixed-effects models to further analyze the 203 intersection genes whose abundances were significantly associated with milk yield (β1 for WGCNA ∩ DEGs, *p* < 0.05, Supplementary Table S7, Figure 5A). The hub genes that were significantly positively associated with milk yield (MY) predominantly included genes such as *HES-6*, *ERN2*, *SHISA2*, *CXCL17*, *IL1RL1*, and *PMP22*, whereas *FBXL22* and *CYP26C1* were more prevalent among the hub genes that were negatively associated with MY (Coefficients > |0.3|, *p* < 0.05, Figure 5A, Supplementary Table S7). We then constructed a random forest machine learning model using these eight hub genes and found that the model can predict milk yield with a correlation coefficient of 0.946 between the predicted and actual values (*p* < 0.001, Figure 5B). The ranking of the importance of hub genes in predicting milk yield is shown in Figure 5C, with *CXCL17*, *FBXL22*, *HES-6*, *SHISA2*, and *ERN2* being the top five most important hub genes. Next, in order to investigate whether the expression patterns of the eight hub genes that have a decisive effect on milk production remain consistent before parturition, we conducted transcriptome sequencing on the liver tissue of dairy cows during the 3 weeks before calving. Remarkably, the expression patterns of these eight hub genes exhibited a high degree of similarity before and after parturition, thereby providing further evidence of their significant role in regulating milk production (Figure 5D).

## 4. Discussion

In this study, we recruited 64 dairy cows and successfully categorized them into high and low MY groups based on their average milk yield during the first three weeks. The continuous monitoring of milk yield showed that cows in the high MY group consistently produced a higher yield than those in the low MY group over the first 3 weeks. Additionally, the difference in average daily milk yield between the high and low MY groups was 11.14 kg/d, thereby further confirming the significant differentiation of the milk yield group categories that we established. Selecting and breeding cows with a higher genetic potential for milk production usually also results in an increased efficiency of converting feed into milk for these animals [24]. Similarly, our study found that high MY cows exhibit higher feed conversion efficiency. Previous studies have also demonstrated that with an increase in milk yield, FCE similarly improves across various lactation stages, from the first through the fourth stage and into the high-production period [25]. This may be attributed to the fact that the enhanced FCE is mainly due to the reduced energy requirements for maintaining vital functions in cows, as the proportion of concentrate in their diet increases and milk yield rises, along with a possible increased mobilization of body tissue reserves for milk production [25].

The liver is pivotal in bolstering the anabolic capabilities of the mammary gland. The net hepatic glucose production, averaging 3.1 kg/day in mid-to-late lactating cows, is sufficient to meet the glucose requirements for milk lactose synthesis and for maintenance purposes [26,27]. Furthermore, the liver exerts a commanding influence in shaping the final quantity and distribution of metabolites available for milk synthesis [27]. Hence, the preservation of hepatic health is imperative for the optimal milk production of dairy cattle. Our results showed that the AST and TBil were significantly lower in High MY cows. Serum AST and TBil are commonly used as indicators of liver function [28]. AST is commonly assessed as an indicator of liver or muscle damage. Any alterations in the serum enzyme activity may be the result of increased activity within cells, particularly hepatocytes, but also indicate structural damage to the cells [29], which could be due to liver dysfunction [30]. Furthermore, when the excretory capacity of liver cells diminishes, blood concentrations of certain metabolites, such as TBil, ammonia, and bile acids, tend to rise [31]. These liver function indicators collectively demonstrated that dairy cows with higher milk production exhibit a healthier liver status under consistent feeding conditions. Consistent with the results of the present study, a positive association between higher milk production and higher liver health status was also reported in several previous studies [32,33]. Although the precise molecular mechanism underlying the relationship between liver health and increased milk production remains unknown, it is widely acknowledged that a robust and healthy liver plays a crucial role in facilitating extensive milk synthesis.

The positive effects of antioxidant supplementation on milk yield were reported widely in previous studies. Evangelista et al. (2022) found that the supplementation of antioxidant ZnSe increased the milk yield by ~3.5% in Buffalo [34]. Matra and Wanapat (2022) demonstrated that the inclusion of a dragon fruit peel pellet could increase the milk yield and enhance the plasma a-diphenyl-b-picrylhydrazyl (DPPH) scavenging activity and antioxidant capacity [35]. Gessner et al. (2015, 2017) observed an increase in milk yield [36] and the inhibition of endoplasmic reticulum stress-induced unfolded protein response and inflammatory processes after feeding grape seed and grape marc meal extract (GSGME). Although TAOC was not significantly increased by GSGME in their study [37], the antioxidant capacity of grape seed was reported in multiple studies performed on diverse animals [38,39,40,41]. Collectively, in this study, the increase in TAOC in the serum of high MY cows further verified the positive relationship between antioxidant capacity and milk production.

The diverse hepatic functions are essential to a cow’s milk production [42], particularly during the critical transition period between three weeks before and three weeks after calving, also called the transition period [43]. Utilizing RNA-seq and bioinformatics analysis methods, we conducted sequencing on 17 postpartum dairy cow liver samples after calving. Our data clearly indicated a strong increase in the expression of genes involved with morphogenesis and development. Interestingly, the genes that intersect with the up-regulated DEGs in WGCNA were also significantly enriched in biological pathways associated with morphogenesis and development in the pathway enrichment analysis. This may be because high-yielding dairy cows need a more active liver growth and development process to sustain high-level lactation. This process is designed to optimize the liver’s structure and function to meet the demands of high milk production and to improve its ability to manage increased energy and nutritional needs, thus facilitating the synthesis of high yield milk. We found that pathways associated with immunity and inflammation are enriched in dairy cows with high milk yield. In the liver, homeostatic inflammatory processes control hemodynamic changes, capillary permeability, leukocyte migration into tissues, and the secretion of inflammatory mediators [44]. Therefore, the immune system of high MY cows was more active, enabling them to meet higher metabolic demands and adapt to more complex environmental conditions. In addition, we note that lipid metabolic process and oxidoreductase activity in liver were increased in high MY cows compared with low MY cows in this study. Previous studies have demonstrated that the primary adaptation in the early postpartum period is metabolic, primarily lipid metabolism, with fatty acid oxidation and mitochondria playing a crucial role [5]. This further demonstrates that high MY cows have a superior ability to utilize gluconeogenesis and non-esterified fatty acids during the peripartum period compared to low MY cows, through the process of oxidation and re-esterification in the liver.

Based on the linear mixed-effects model, we identified six promising hub genes promoting milk production and two negative hub genes. Among them, *CXCL17* ranks first as a hub gene in the prediction of milk production. *CXCL-17* is integral to maintaining homeostasis across various mucosal interfaces, involving the regulation of myeloid-cell recruitment, the promotion of angiogenesis, and the management of microbial populations [45]. In addition, the expression level of *CXCL17* in the ruminant liver is relatively high [46], and according to the results of related studies [47,48], we speculated that *CXCL17* may be related to the immune function and cell proliferation of cow liver. Therefore, as a chemokine, *CXCL17* recruits immune cells and may play a significant role in the immune surveillance and inflammatory response within the livers of high MY cows. Importantly, high MY cows require more energy and nutrients to support their high milk production. The liver, serving as the central hub of metabolism, may activate cell proliferation pathways to increase the number of liver cells, thereby enhancing its capacity to process nutrients and meet the elevated metabolic demands. This hypothesis is actually supported by a previous study where the process or pathway involved in the cell cycle and cell mitosis were activated during lactation initiation [5]. In addition, the activated cell cycle and cell proliferation biological process were also observed in the liver of cows fed GSGME, which was confirmed to significantly increase the milk yield by ~10.2% in 2 to 9 weeks of lactation [36,37]. The findings from the present study, along with previous research, collectively indicate a crucial role of activated cell proliferation in the bovine liver for initiating lactation and sustaining high milk production. Unfortunately, our analysis of prepartum liver data from high MY cows revealed that the expression pattern of the *CXCL17* gene before and after parturition showed no significant similarity; specifically, the prepartum expression level of *CXCL17* was lower than that of the low MY group. These differences could be attributed to changes in the immune status between the prepartum and postpartum phases. Research indicates that immune-related pathways are activated following parturition, in contrast to the prepartum state [5]. Consequently, high-yielding dairy cows might have unique immune regulatory mechanisms prior to parturition that are designed to enhance their production efficiency.

Moreover, the genes *HES6* [49,50], *SHISA2* [51], *PMP22* [52], and *IL1RL1* [53] are primarily associated with the progression of cancer and diseases. This suggests a significant amount of research on these genes in humans and mice, but there is a scarcity of definitive studies or reports on their roles in ruminant animals. In the current genetic research, we have not yet identified any hub genes with a direct correlation. We are committed to further investigation and the execution of related experiments to substantiate any potential connections. As of now, we cannot definitively conclude the absence of a relationship. While comprehensive research reports specifically addressing this area have yet to emerge, it is well established that numerous genes are pivotal in the initiation and progression of cancer. Regarding the links to milk production and liver function, no corresponding research findings have been brought to light thus far.

## 5. Conclusions

The current research highlights the pivotal function of the liver in influencing milk production in dairy cows. The enriched morphogenetic and developmental processes within the liver are likely linked to superior milk production. Additionally, the eight key genes identified in our investigation could serve as valuable genetic resources for future breeding programs in dairy cattle. The expression patterns of these eight hub genes remained remarkably consistent before and after parturition, further confirming the pivotal role of the hub genes in milk production. *CXCL17*, which demonstrated the highest contribution in the random forest machine learning model for predicting milk yield, holds significant potential for future research.

## Data Availability

The raw sequence data reported in this study have been deposited in the Genome Sequence Archive in the Beijing Institute of Genomics (BIG) Data Center (National Genomics Data Center Members and Partners, 2020), Chinese Academy of Sciences, under accession numbers CRA002550, publicly accessible at https://bigd.big.ac.cn/gsa, accessed on 17 February 2020.

## Supplementary Materials

The following supporting information can be downloaded at: https://www.mdpi.com/article/10.3390/genes15091229/s1, Table S1: Ingredient composition of diets for cows before and after parturition; Table S2: Schematic diagram of milk yield groupings; Table S3: GO enrichment analysis was conducted on the DEGs in the high MY group; Table S4: GO enrichment analysis was conducted on the DEGs in the low MY group; Table S5: KEGG enrichment analysis was conducted on the DEGs in the high MY group; Table S6: GO terms associated with the 77 intersection genes where WGCNA overlapped with up-regulated DGEs; Table S7: Relationship between intersection genes and milk yield described in Figure 5A.
