# Supplementary material for: Hepatic Transcriptome Reveals Potential Key Genes Contributing to Differential Milk Production
Source: Genes (Basel). 2024 Sep 20;15(9):1229. doi: 10.3390/genes15091229 (PMC11431119; doi:10.3390/genes15091229)
Supplement: Supplementary file 1 [file genes-15-01229-s001.zip › genes-3206766-supplementary.pdf]

Supplementary Materials

Table S1. Ingredient composition of diets for cows before and after parturition

Ingredient (%)	Close-up	Fresh
Grass hay	19.5	-
Oats hay	21.9	4.90
Alfalfa	-	20.0
Corn silage	22.4	22.8
Steam-flaked corn	0	8.9
Cornmeal	5.0	9.4
Soybean meal	3.7	12.4
Cottonseed meal	3.0	-
DDGS[1]	2.6	-
Extracted soybean	-	-
Canola meal solvent	4.7	2.5
Molasses cane	-	1.9
Corn gluten meal	1.3	1.5
Brewers grains	10.1	6.1
Mineral premix[2]	4.8	3.7
Choline	0.4	0.1
BergaFat100[3]	-	0.8
$KHCO_3$	-	0.3
MT-BOND[4]	-	0.1
Optigen-slow[5]	-	0.2
Diamond V-XPC Yeast product[6]	0.2	0.1
Glycoline[7]	-	1.6
B vitamin	0.4	0.2

[1]DDGS = distillers dried grains with solubles; nutrient-rich by-product of dry-milled ethanol production.

[2]Mineral premix: Ca, P, Mg, K, Na, Cl, and S.

[3]BergaFat100 (Berg and Schmidt Nutrition Sdn. Bhd., Malaysia): bypass fats for ruminants providing extra energy without a carrier.

[4]MinTech (Tianjin) Minerals Co. Ltd., Tianjin, China.

[5]Nonprotein nitrogen source for ruminants (Alltech Inc., Nicholasville, KY).

[6]Diamond V XP yeast culture supplement (FD00365CHN-XP, Diamond V, Cedar Rapids, IA).

[7]Vitalac Co. Ltd., Carnoët, France.

Table S2. Schematic diagram of milk yield groupings.

Cow ID	Average milk yield of first 3 week after calving	Liver sample	Group	Liver sample before calving	Liver sample after calving
02121530	54.4	No	High		
04120147	53.0	No	High		
04120316	53.5	No	High		
04120339	50.8	Yes	High	T1_3B	T1_3A
04121646	60.8	No	High		
04121772	57.1	Yes	High	T2_4B	T2_4A
04122013	54.9	No	High		
04122552	54.7	No	High		
04122605	57.2	No	High		
04122783	54.6	Yes	High	T1_2B	T1_2A
04122846	55.2	Yes	High	T1_1B	T1_1A
04123159	54.9	No	High		
04123781	50.7	Yes	High	T1_5B	
04124590	50.4	No	High		
04124817	57.6	Yes	High	T1_4B	T1_4A
2122733	54.3	No	High		
2123230	53.4	No	High		
2133683	49.1	No	High		
4120135	52.2	No	High		
4120614	49.6	No	High		
4121070	53.0	No	High		
4121389	50.3	No	High		
4121425	52.9	No	High		
4121663	49.1	No	High		
4122230	54.5	No	High		
4122607	51.1	No	High		
4124019	53.8	Yes	High	T2_2B	T2_2A
4124165	49.2	No	High		
4124246	57.2	No	High		
4124346	54.4	No	High		
4124598	56.1	No	High		
4124840	52.0	No	High		
4125013	55.7	No	High		
02121246	44.3	No	Low		
02122778	48.3	No	Low		
02123264	39.5	No	Low		
04120361	39.7	Yes	Low	T2_3B	T2_3A
04120588	37.9	No	Low		
04120616	32.3	No	Low		
04121017	45.1	No	Low		
04121848	44.3	Yes	Low	T2_1B	T2_1A
04121897	42.6	No	Low		
04121976	40.4	No	Low		
04122215	44.3	No	Low		

04124308	37.2	Yes	Low	T2_5B	T2_5A
2121256	44.8	Yes	Low	T3_2B	T3_2A
2122639	37.9	No	Low		
2122680	37.6	Yes	Low	T3_3B	T3_3A
2123479	47.7	No	Low		
2123482	37.9	No	Low		
4120106	43.2	Yes	Low	T4_3B	T4_3A
4122294	39.8	Yes	Low	T4_4B	T4_4A
4122319	41.5	Yes	Low	T3_4B	T3_4A
4123234	45.5	Yes	Low	T4_2B	T4_2A
4123287	45.9	No	Low		
4123975	43.6	No	Low		
4124120	44.2	Yes	Low	T4_1B	T4_1A
4124333	42.9	No	Low		
4124886	45.6	Yes	Low	T3_1B	T3_1A
4124889	37.6	No	Low		
4125623	48.2	No	Low		
4125786	39.5	No	Low		
4125808	47.9	No	Low		
4125819	47.9	No	Low		

Table S3. GO enrichment analysis was conducted on the DEGs in the high MY group.

ID	Description	Count	GeneRatio	*p*-value	*p* adjust	q-value
GO:0009653	anatomical structure morphogenesis	24	0.24	9.53148E-05	0.009537439	0.00863477
GO:0006629	lipid metabolic process	21	0.21	7.72754E-05	0.008247865	0.007467248
GO:0016491	oxidoreductase activity	17	0.191011	0.000114105	0.02692875	0.025583514
GO:0051240	positive regulation of multicellular organismal process	18	0.18	5.84769E-05	0.007801791	0.007063392
GO:0035239	tube morphogenesis	16	0.16	8.56383E-07	0.00137107	0.001241305
GO:0035295	tube development	16	0.16	1.18789E-05	0.002860748	0.002589993
GO:0048646	anatomical structure formation involved in morphogenesis	16	0.16	1.42948E-05	0.002860748	0.002589993
GO:2000026	regulation of multicellular organismal development	16	0.16	7.20955E-05	0.008247865	0.007467248
GO:0072359	circulatory system development	15	0.15	5.52457E-05	0.007801791	0.007063392
GO:0001568	blood vessel development	14	0.14	6.14255E-06	0.002854621	0.002584446
GO:0001944	vasculature development	14	0.14	8.3521E-06	0.002854621	0.002584446
GO:0048514	blood vessel morphogenesis	13	0.13	6.98626E-06	0.002854621	0.002584446
GO:0006954	inflammatory response	13	0.13	0.000247015	0.017975969	0.016274637
GO:0001525	angiogenesis	12	0.12	8.91512E-06	0.002854621	0.002584446
GO:0032787	monocarboxylic acid metabolic process	12	0.12	0.000440471	0.03066061	0.027758743
GO:0019216	regulation of lipid metabolic process	10	0.1	1.31914E-05	0.002860748	0.002589993
GO:0071363	cellular response to growth factor stimulus	10	0.1	0.000157773	0.014033067	0.01270491
GO:0070848	response to growth factor	10	0.1	0.000229139	0.017469141	0.015815778
GO:0016042	lipid catabolic process	8	0.08	0.000847323	0.04677807	0.042350769
GO:0045765	regulation of angiogenesis	7	0.07	0.000169017	0.014241938	0.012894013
GO:1901342	regulation of vasculature development	7	0.07	0.000214367	0.017160054	0.015535944
GO:0045766	positive regulation of angiogenesis	6	0.06	3.02815E-05	0.004848063	0.004389219
GO:1904018	positive regulation of vasculature development	6	0.06	3.02815E-05	0.004848063	0.004389219
GO:0120254	olefinic compound metabolic process	5	0.05	0.000590641	0.036076465	0.032662016
GO:0001954	positive regulation of cell-matrix adhesion	4	0.04	7.42029E-05	0.008247865	0.007467248
GO:0035924	cellular response to vascular endothelial growth factor	4	0.04	0.000109362	0.010299298	0.009324523

	stimulus					
GO:0044344	cellular response to fibroblast growth factor stimulus	4	0.04	0.000481583	0.032125613	0.029085091
GO:0001952	regulation of cell-matrix adhesion	4	0.04	0.00060841	0.036076465	0.032662016
GO:0071774	response to fibroblast growth factor	4	0.04	0.00060841	0.036076465	0.032662016
GO:0010811	positive regulation of cell-substrate adhesion	4	0.04	0.000757258	0.043298945	0.039200925

Table S4. GO enrichment analysis was conducted on the DEGs in the low MY group.

ID	Description	Count	GeneRatio	*p*-value	*p* adjust	q-value
GO:0016323	Basolateral plasma membrane	7	0.06034483	0.000284649	0.02446264	0.0236043
GO:0009925	Basal plasma membrane	7	0.06034483	0.000320434	0.02446264	0.0236043
GO:0045178	Basal part of cell	7	0.06034483	0.000359745	0.02446264	0.0236043

Table S5. KEGG enrichment analysis was conducted on the DEGs in the high MY group.

ID	Description	Count	GeneRatio	*p*-value	*p* adjust	q-value
bta05171	Coronavirus disease - COVID-19	15	0.08152174	0.000340188	0.008535632	0.007357181
bta04060	Cytokine-cytokine receptor interaction	15	0.08152174	0.002078133	0.034023178	0.029325852
bta04062	Chemokine signaling pathway	13	0.07065217	8.36672E-05	0.005143145	0.004433069
bta04061	Viral protein interaction with cytokine and cytokine receptor	10	0.05434783	1.35216E-05	0.001269259	0.001094022
bta05142	Chagas disease	10	0.05434783	0.000104551	0.005143145	0.004433069
bta00830	Retinol metabolism	9	0.04891304	9.83068E-06	0.001269259	0.001094022
bta00140	Steroid hormone biosynthesis	9	0.04891304	1.37963E-05	0.001269259	0.001094022
bta05323	Rheumatoid arthritis	9	0.04891304	0.00016027	0.005529324	0.004765931
bta05133	Pertussis	8	0.04347826	0.000119017	0.005143145	0.004433069
bta05140	Leishmaniasis	8	0.04347826	0.000130442	0.005143145	0.004433069
bta04146	Peroxisome	8	0.04347826	0.000202012	0.006195022	0.005339721
bta04620	Toll-like receptor signaling pathway	8	0.04347826	0.002218903	0.034023178	0.029325852
bta05204	Chemical carcinogenesis - DNA adducts	7	0.03804348	0.000254871	0.007034443	0.00606325
bta04657	IL-17 signaling pathway	7	0.03804348	0.002197233	0.034023178	0.029325852
bta00982	Drug metabolism - cytochrome P450	6	0.0326087	0.00125309	0.026604057	0.022931033
bta00980	Metabolism of xenobiotics by cytochrome P450	6	0.0326087	0.001877605	0.034023178	0.029325852
bta00040	Pentose and glucuronate interconversions	5	0.02717391	0.000404976	0.009314448	0.008028472
bta00053	Ascorbate and aldarate metabolism	4	0.02173913	0.001979588	0.034023178	0.029325852
bta00052	Galactose metabolism	4	0.02173913	0.002907029	0.042228416	0.036398253

Table S6 GO terms associated with the 77 intersection genes where WGCNA overlapped with up-regulated DGEs.

ID	Description	Count	GeneRatio	*p*-value	*p* adjust	q-value
GO:0051240	Positive Regulation of Multicellular Organismal Process	13	0.48148148	2.4707E-09	2.1619E-06	1.6203E-06
GO:0001819	Positive Regulation of Cytokine Production	8	0.2962963	2.3911E-07	0.00010461	7.8402E-05
GO:0006954	Inflammatory Response	9	0.33333333	8.4339E-07	0.0002143	0.00016061
GO:0060326	Cell Chemotaxis	6	0.22222222	9.7963E-07	0.0002143	0.00016061
GO:0001568	Blood Vessel Development	8	0.2962963	2.0847E-06	0.00027803	0.00020838
GO:0001817	Regulation of Cytokine Production	8	0.2962963	2.0847E-06	0.00027803	0.00020838
GO:0001816	Cytokine Production	8	0.2962963	2.542E-06	0.00027803	0.00020838
GO:0001944	Vasculature Development	8	0.2962963	2.542E-06	0.00027803	0.00020838
GO:0001525	Angiogenesis	7	0.25925926	4.0758E-06	0.00039007	0.00029235
GO:0035239	Tube Morphogenesis	8	0.2962963	4.6657E-06	0.00039007	0.00029235
GO:0050920	Regulation of Chemotaxis	5	0.18518519	4.9037E-06	0.00039007	0.00029235
GO:2000026	Regulation Of Multicellular Organismal Development	9	0.33333333	6.7089E-06	0.00048919	0.00036664
GO:0010557	Positive Regulation of Macromolecule Biosynthetic Process	12	0.44444444	7.3304E-06	0.00049339	0.00036978
GO:0010628	Positive Regulation of Gene Expression	9	0.33333333	8.5241E-06	0.00053275	0.00039928
GO:0048514	Blood Vessel Morphogenesis	7	0.25925926	1.0231E-05	0.00059543	0.00044626
GO:0031328	Positive Regulation of Cellular Biosynthetic Process	12	0.44444444	1.0888E-05	0.00059543	0.00044626
GO:0009891	Positive Regulation of Biosynthetic Process	12	0.44444444	1.2115E-05	0.00062358	0.00046735
GO:0045765	Regulation of Angiogenesis	5	0.18518519	1.4017E-05	0.00068138	0.00051068
GO:1901342	Regulation of Vasculature Development	5	0.18518519	1.685E-05	0.00071756	0.0005378
GO:0006935	Chemotaxis	6	0.22222222	1.6973E-05	0.00071756	0.0005378
GO:0022603	Regulation of Anatomical Structure Morphogenesis	7	0.25925926	1.7348E-05	0.00071756	0.0005378
GO:0042330	Taxis	6	0.22222222	1.8042E-05	0.00071756	0.0005378
GO:0035295	Tube Development	8	0.2962963	2.0421E-05	0.000736	0.00055162
GO:0045766	Positive Regulation of Angiogenesis	4	0.14814815	2.1076E-05	0.000736	0.00055162
GO:1904018	Positive Regulation of Vasculature Development	4	0.14814815	2.1076E-05	0.000736	0.00055162
GO:0072359	Circulatory System Development	8	0.2962963	2.1925E-05	0.000736	0.00055162

GO:0048646	Anatomical Structure Formation Involved in Morphogenesis	8	0.2962963	2.2711E-05	0.000736	0.00055162
GO:0048246	Macrophage Chemotaxis	3	0.11111111	2.4252E-05	0.00075788	0.00056801
GO:0001935	Endothelial Cell Proliferation	4	0.14814815	5.2332E-05	0.00156756	0.00117484
GO:0043068	Positive Regulation of Programmed Cell Death	6	0.22222222	5.3745E-05	0.00156756	0.00117484
GO:0006952	Defense Response	10	0.37037037	6.2174E-05	0.00175492	0.00131526
GO:0032101	Regulation of Response to External Stimulus	8	0.2962963	6.7867E-05	0.00185574	0.00139083
GO:1905517	Macrophage Migration	3	0.11111111	7.2365E-05	0.00191876	0.00143806
GO:0050793	Regulation of Developmental Process	10	0.37037037	0.00012149	0.00312668	0.00234336
GO:0032731	Positive Regulation of Interleukin-1 Beta Production	3	0.11111111	0.00013353	0.00324553	0.00243244
GO:0044344	Cellular Response to Fibroblast Growth Factor Stimulus	3	0.11111111	0.00013353	0.00324553	0.00243244
GO:0030595	Leukocyte Chemotaxis	4	0.14814815	0.00015572	0.00367453	0.00275396
GO:0071774	Response to Fibroblast Growth Factor	3	0.11111111	0.00015958	0.00367453	0.00275396
GO:0097529	Myeloid Leukocyte Migration	4	0.14814815	0.00019965	0.00447922	0.00335705
GO:0032732	Positive Regulation of Interleukin-1 Production	3	0.11111111	0.00022112	0.00483695	0.00362517
GO:0002682	Regulation of Immune System Process	8	0.2962963	0.00027957	0.00588941	0.00441396
GO:0009653	Anatomical Structure Morphogenesis	10	0.37037037	0.00028269	0.00588941	0.00441396
GO:0032611	Interleukin-1 Beta Production	3	0.11111111	0.00029626	0.00589152	0.00441554
GO:0032651	Regulation of Interleukin-1 Beta Production	3	0.11111111	0.00029626	0.00589152	0.00441554
GO:0002688	Regulation of Leukocyte Chemotaxis	3	0.11111111	0.00043705	0.00839029	0.0062883
GO:0043065	Positive Regulation of Apoptotic Process	5	0.18518519	0.00045068	0.00839029	0.0062883
GO:0071363	Cellular Response to Growth Factor Stimulus	5	0.18518519	0.00045068	0.00839029	0.0062883
GO:0002376	Immune System Process	10	0.37037037	0.00046267	0.00842966	0.00631781
GO:0001936	Regulation of Endothelial Cell Proliferation	3	0.11111111	0.00049203	0.00842966	0.00631781
GO:0032612	Interleukin-1 Production	3	0.11111111	0.00049203	0.00842966	0.00631781
GO:0032652	Regulation of Interleukin-1 Production	3	0.11111111	0.00049203	0.00842966	0.00631781
GO:0040011	Locomotion	7	0.25925926	0.00050096	0.00842966	0.00631781
GO:0071621	Granulocyte Chemotaxis	3	0.11111111	0.00055127	0.00882127	0.00661131
GO:0071675	Regulation of Mononuclear Cell Migration	3	0.11111111	0.00055127	0.00882127	0.00661131

GO:0070848	Response to Growth Factor	5	0.18518519	0.00055448	0.00882127	0.00661131
GO:0042127	Regulation Cell Population Proliferation	8	0.2962963	0.00074654	0.01160238	0.00869568
GO:0050921	Positive Regulation of Chemotaxis	3	0.11111111	0.00075581	0.01160238	0.00869568
GO:0016477	Cell Migration	7	0.25925926	0.00082625	0.01246494	0.00934214
GO:0008285	Negative Regulation of Cell Population Proliferation	5	0.18518519	0.00084561	0.01254082	0.00939901
GO:0008219	Cell Death	9	0.33333333	0.00088102	0.01263758	0.00947153
GO:0012501	Programmed Cell Death	9	0.33333333	0.00088102	0.01263758	0.00947153
GO:0040012	Regulation of Locomotion	6	0.22222222	0.00107444	0.01516349	0.01136464
GO:0097530	Granulocyte Migration	3	0.11111111	0.00119368	0.01657893	0.01242547
GO:2001141	Regulation of RNA Biosynthetic Process	9	0.33333333	0.00129386	0.01745786	0.01308421
GO:0070374	Positive Regulation of Erk1 and Erk2 Cascade	3	0.11111111	0.00129687	0.01745786	0.01308421
GO:0050900	Leukocyte Migration	4	0.14814815	0.00137017	0.01816518	0.01361432
GO:0071674	Mononuclear Cell Migration	3	0.11111111	0.00140552	0.01835572	0.01375713
GO:0050727	Regulation of Inflammatory Response	4	0.14814815	0.00143439	0.01845722	0.0138332
GO:0009719	Response to Endogenous Stimulus	7	0.25925926	0.00147148	0.01866011	0.01398526
GO:0032102	Negative Regulation of Response to External Stimulus	4	0.14814815	0.00156911	0.01908896	0.01430667
GO:0009595	Detection of Biotic Stimulus	2	0.07407407	0.00159256	0.01908896	0.01430667
GO:0010573	Vascular Endothelial Growth Factor Production	2	0.07407407	0.00159256	0.01908896	0.01430667
GO:0150117	Positive Regulation of Cell-Substrate Junction Organization	2	0.07407407	0.00159256	0.01908896	0.01430667
GO:0043067	Regulation of Programmed Cell Death	7	0.25925926	0.0017243	0.02013558	0.01509109
GO:0051094	Positive Regulation of Developmental Process	6	0.22222222	0.00172591	0.02013558	0.01509109
GO:0002683	Negative Regulation of Immune System Process	4	0.14814815	0.00178743	0.02057898	0.0154234
GO:0048870	Cell Motility	7	0.25925926	0.00193546	0.02094694	0.01569918
GO:0001954	Positive Regulation of Cell-Matrix Adhesion	2	0.07407407	0.00193909	0.02094694	0.01569918
GO:0035914	Skeletal Muscle Cell Differentiation	2	0.07407407	0.00193909	0.02094694	0.01569918
GO:0051893	Regulation of Focal Adhesion Assembly	2	0.07407407	0.00193909	0.02094694	0.01569918
GO:0090109	Regulation of Cell-Substrate Junction Assembly	2	0.07407407	0.00193909	0.02094694	0.01569918
GO:0010647	Positive Regulation of Cell Communication	7	0.25925926	0.00212651	0.02269137	0.01700659

GO:0023056	Positive Regulation of Signaling	7	0.25925926	0.00216643	0.02283889	0.01711715
GO:0035924	Cellular Response to Vascular Endothelial Growth Factor Stimulus	2	0.07407407	0.00231809	0.02414673	0.01809734
GO:0050673	Epithelial Cell Proliferation	4	0.14814815	0.00266768	0.0274486	0.02057201
GO:0048041	Focal Adhesion Assembly	2	0.07407407	0.00272918	0.0274486	0.02057201
GO:1904035	Regulation of Epithelial Cell Apoptotic Process	2	0.07407407	0.00272918	0.0274486	0.02057201
GO:0006915	Apoptotic Process	8	0.2962963	0.00293864	0.02921947	0.02189922
GO:0036230	Granulocyte Activation	2	0.07407407	0.00317198	0.03017626	0.02261632
GO:0042698	Ovulation Cycle	2	0.07407407	0.00317198	0.03017626	0.02261632
GO:0150116	Regulation of Cell-Substrate Junction Organization	2	0.07407407	0.00317198	0.03017626	0.02261632
GO:0002274	Myeloid Leukocyte Activation	3	0.11111111	0.00317282	0.03017626	0.02261632
GO:0002685	Regulation of Leukocyte Migration	3	0.11111111	0.00336155	0.03162745	0.02370394
GO:0070372	Regulation of Erk1 and Erk2 Cascade	3	0.11111111	0.00355705	0.03255459	0.02439881
GO:0031347	Regulation of Defense Response	5	0.18518519	0.00358505	0.03255459	0.02439881
GO:0071495	Cellular Response to Endogenous Stimulus	6	0.22222222	0.00360208	0.03255459	0.02439881
GO:0007044	Cell-Substrate Junction Assembly	2	0.07407407	0.00364611	0.03255459	0.02439881
GO:0050848	Regulation of Calcium-Mediated Signaling	2	0.07407407	0.00364611	0.03255459	0.02439881
GO:0045944	Positive Regulation of Transcription by RNA Polymerase II	5	0.18518519	0.00395684	0.03472604	0.02602625
GO:0002237	Response to Molecule of Bacterial Origin	3	0.11111111	0.00396869	0.03472604	0.02602625
GO:0032757	Positive Regulation of Interleukin-8 Production	2	0.07407407	0.00415121	0.03533025	0.02647909
GO:0071347	Cellular Response to Interleukin-1	2	0.07407407	0.00415121	0.03533025	0.02647909
GO:0048584	Positive Regulation of Response to Stimulus	8	0.2962963	0.00415888	0.03533025	0.02647909
GO:0030334	Regulation of Cell Migration	5	0.18518519	0.00435578	0.03638961	0.02727306
GO:0043542	Endothelial Cell Migration	3	0.11111111	0.00440834	0.03638961	0.02727306
GO:0070371	Erk1 and Erk2 Cascade	3	0.11111111	0.00440834	0.03638961	0.02727306
GO:0016525	Negative Regulation of Angiogenesis	2	0.07407407	0.0046869	0.03661644	0.02744306
GO:0032637	Interleukin-8 Production	2	0.07407407	0.0046869	0.03661644	0.02744306
GO:0032677	Regulation of Interleukin-8 Production	2	0.07407407	0.0046869	0.03661644	0.02744306
GO:0071677	Positive Regulation of Mononuclear Cell Migration	2	0.07407407	0.0046869	0.03661644	0.02744306

GO:1904019	Epithelial Cell Apoptotic Process	2	0.07407407	0.0046869	0.03661644	0.02744306
GO:2000181	Negative Regulation of Blood Vessel Morphogenesis	2	0.07407407	0.0046869	0.03661644	0.02744306
GO:0051241	Negative Regulation of Multicellular Organismal Process	5	0.18518519	0.00489431	0.03789839	0.02840384
GO:0040017	Positive Regulation of Locomotion	4	0.14814815	0.00508936	0.03862368	0.02894743
GO:0001938	Positive Regulation of Endothelial Cell Proliferation	2	0.07407407	0.00525282	0.03862368	0.02894743
GO:0001952	Regulation of Cell-Matrix Adhesion	2	0.07407407	0.00525282	0.03862368	0.02894743
GO:0150115	Cell-Substrate Junction Organization	2	0.07407407	0.00525282	0.03862368	0.02894743
GO:1901343	Negative Regulation of Vasculature Development	2	0.07407407	0.00525282	0.03862368	0.02894743
GO:2000401	Regulation of Lymphocyte Migration	2	0.07407407	0.00525282	0.03862368	0.02894743
GO:0006355	Regulation of DNA-Templated Transcription	8	0.2962963	0.00531164	0.03871159	0.02901332
GO:0045893	Positive Regulation of DNA-Templated Transcription	6	0.22222222	0.00535326	0.03871159	0.02901332
GO:1902680	Positive Regulation of RNA Biosynthetic Process	6	0.22222222	0.00545002	0.03908825	0.02929561
GO:0044403	Biological Process Involved in Symbiotic Interaction	3	0.11111111	0.00563366	0.04007684	0.03003654
GO:0044419	Biological Process Involved in Interspecies Interaction Between Organisms	7	0.25925926	0.00568896	0.04008526	0.03004285
GO:2000145	Regulation of Cell Motility	5	0.18518519	0.00572647	0.04008526	0.03004285
GO:0002690	Positive Regulation of Leukocyte Chemotaxis	2	0.07407407	0.0058486	0.04029545	0.03020038
GO:0010811	Positive Regulation of Cell-Substrate Adhesion	2	0.07407407	0.0058486	0.04029545	0.03020038
GO:0051726	Regulation of Cell Cycle	5	0.18518519	0.0059817	0.04089056	0.0306464
GO:1902533	Positive Regulation of Intracellular Signal Transduction	5	0.18518519	0.00611232	0.04145955	0.03107284
GO:0019233	Sensory Perception of Pain	2	0.07407407	0.00647387	0.04270116	0.03200339
GO:0030593	Neutrophil Chemotaxis	2	0.07407407	0.00647387	0.04270116	0.03200339
GO:0045670	Regulation of Osteoclast Differentiation	2	0.07407407	0.00647387	0.04270116	0.03200339
GO:0009967	Positive Regulation of Signal Transduction	6	0.22222222	0.00649058	0.04270116	0.03200339
GO:0042981	Regulation of Apoptotic Process	6	0.22222222	0.00660216	0.04311113	0.03231065
GO:0051928	Positive Regulation of Calcium Ion Transport	2	0.07407407	0.00712829	0.04620187	0.03462709
GO:0002684	Positive Regulation of Immune System Process	5	0.18518519	0.00723144	0.04652581	0.03486987
GO:0001775	Cell Activation	5	0.18518519	0.00753244	0.04810863	0.03605615
GO:0006357	Regulation Of Transcription by RNA Polymerase II	6	0.22222222	0.00767119	0.04852082	0.03636508

GO:0070555	Response to Interleukin-1	2	0.07407407	0.00781149	0.04852082	0.03636508
GO:1901890	Positive Regulation of Cell Junction Assembly	2	0.07407407	0.00781149	0.04852082	0.03636508
GO:0060284	Regulation of Cell Development	4	0.14814815	0.00781878	0.04852082	0.03636508
GO:0048513	Animal Organ Development	8	0.2962963	0.00796275	0.0488603	0.03661951
GO:0050678	Regulation of Epithelial Cell Proliferation	3	0.11111111	0.00798517	0.0488603	0.03661951
GO:0051254	Positive Regulation of RNA Metabolic Process	6	0.22222222	0.00818502	0.04973537	0.03727535
GO:0033993	Response to Lipid	4	0.14814815	0.00824352	0.04974536	0.03728284
GO:0020037	Heme Binding	3	0.13636364	0.00103373	0.03493006	0.02886128
GO:0046906	Tetrapyrrole Binding	3	0.13636364	0.00162541	0.03493006	0.02886128
GO:0003690	Double-Stranded DNA Binding	4	0.18181818	0.00176155	0.03493006	0.02886128
GO:0042379	Chemokine Receptor Binding	2	0.09090909	0.00200081	0.03493006	0.02886128
GO:0008201	Heparin Binding	3	0.13636364	0.00211756	0.03493006	0.02886128
GO:0005126	Cytokine Receptor Binding	3	0.13636364	0.00225355	0.03493006	0.02886128

Table S7. Relationship between intersection genes and milk yield described in Figure 5A.

Ensembl gene ID	Coefficients	Standard error	t-value	*p*-value	Direction of association with MY	Lable	Gene name
ENSBTAG00000019070	0.32550659	0.12860387	2.53107923	0.02304573	Positive	ENSBTAG00000019070	peripheral myelin protein 22(PMP22)
ENSBTAG00000018652	0.40787183	0.1353495	3.01347139	0.0087299	Positive	ENSBTAG00000018652	C-X-C motif chemokine ligand 17(CXCL17)
ENSBTAG00000018571	0.38677623	0.16947978	2.28213792	0.03749533	Positive	ENSBTAG00000018571	interleukin 1 receptor like 1(IL1RL1)
ENSBTAG00000018214	0.43769648	0.18007811	2.43059241	0.02809121	Positive	ENSBTAG00000018214	shisa family member 2(SHISA2)
ENSBTAG00000014456	0.6296518	0.21084063	2.98638736	0.00922486	Positive	ENSBTAG00000014456	endoplasmic reticulum to nucleus signaling 2(ERN2)
ENSBTAG00000009870	0.76136586	0.23054772	3.30242208	0.00483484	Positive	ENSBTAG00000009870	
ENSBTAG00000043570	0.00010614	3.7936E-05	2.79800834	0.01351392	Positive		
ENSBTAG00000001265	0.0006236	0.0002326	2.68103784	0.01709623	Positive		
ENSBTAG00000010841	0.0008916	0.00025122	3.54912477	0.00291446	Positive		
ENSBTAG00000013054	0.00445765	0.00141795	3.14372404	0.0066917	Positive		
ENSBTAG00000002902	0.01171329	0.00486089	2.40969977	0.02926454	Positive		
ENSBTAG00000020148	0.01348917	0.00562404	2.39848251	0.02991351	Positive		
ENSBTAG00000040128	0.01383146	0.00568676	2.43222046	0.02800167	Positive		
ENSBTAG00000047739	0.01596689	0.00604474	2.64145317	0.01850461	Positive		
ENSBTAG00000004997	0.02695877	0.01098782	2.45351346	0.02685534	Positive		
ENSBTAG00000020350	0.03485848	0.01395213	2.49843423	0.02458165	Positive		
ENSBTAG00000003176	0.0395969	0.01518253	2.60805632	0.01977914	Positive		
ENSBTAG00000037929	0.04074781	0.01850444	2.20205579	0.04372367	Positive		

ENSBTAG00000010637	0.04321212	0.01680766	2.57097717	0.02129297	Positive		
ENSBTAG00000034206	0.05530842	0.02183043	2.53354644	0.02293346	Positive		
ENSBTAG00000018260	0.06086361	0.02165201	2.81099161	0.01316443	Positive		
ENSBTAG00000001879	0.07673748	0.02134241	3.59553886	0.00264979	Positive		
ENSBTAG00000009579	0.09328263	0.02391287	3.90093794	0.00141851	Positive		
ENSBTAG00000046837	0.11724757	0.04395227	2.66761105	0.01756194	Positive		
ENSBTAG00000010119	0.12302958	0.04177426	2.94510519	0.01003272	Positive		
ENSBTAG00000015885	0.13064107	0.05481122	2.38347306	0.0308032	Positive		
ENSBTAG00000002937	0.15974584	0.06036332	2.64640567	0.01832247	Positive		
ENSBTAG00000008154	0.16875175	0.07107548	2.37426094	0.03136157	Positive		
ENSBTAG00000034147	0.22422449	0.09702523	2.31099154	0.03546237	Positive		
ENSBTAG00000008857	0.22965074	0.06847512	3.35378351	0.00435144	Positive		
ENSBTAG00000039973	-0.67519949	0.2759045	-2.44722172	0.02718934	Negative	ENSBTAG00000039973	cytochrome P450 family 26 subfamily C member 1(CYP26C1)
ENSBTAG00000008807	-0.31269644	0.0950988	-3.28812181	0.00497869	Negative	ENSBTAG00000008807	F-box and leucine rich repeat protein 22(FBXL22)
ENSBTAG00000012608	-0.22803459	0.08521037	-2.67613671	0.01726483	Negative		
ENSBTAG00000002848	-0.22701929	0.04835299	-4.69504135	0.00028752	Negative		
ENSBTAG00000020824	-0.22187178	0.09147031	-2.42561527	0.02836661	Negative		

ENSBTAG00000010185	-0.20588521	0.06845509	-3.00759532	0.00883502	Negative
ENSBTAG00000017956	-0.20577402	0.09489687	-2.16839637	0.04661855	Negative
ENSBTAG00000016742	-0.19457075	0.08252066	-2.35784277	0.0323805	Negative
ENSBTAG00000047092	-0.19357083	0.07265045	-2.6644135	0.01767464	Negative
ENSBTAG00000012761	-0.18603657	0.06850571	-2.71563602	0.01595049	Negative
ENSBTAG00000006966	-0.17818988	0.05575121	-3.19616141	0.0060109	Negative
ENSBTAG00000039871	-0.17334337	0.06825465	-2.5396564	0.02265766	Negative
ENSBTAG00000021202	-0.17282079	0.0695102	-2.48626526	0.02517879	Negative
ENSBTAG00000013177	-0.1690892	0.04981832	-3.39411671	0.00400585	Negative
ENSBTAG00000019885	-0.16511694	0.06980429	-2.36542695	0.031906	Negative
ENSBTAG00000044208	-0.16134995	0.06931656	-2.32772592	0.0343312	Negative
ENSBTAG00000019014	-0.14425809	0.05988319	-2.40899153	0.02930512	Negative
ENSBTAG00000022382	-0.1414113	0.04439301	-3.18544082	0.00614427	Negative

ENSBTAG00000005884	-0.14075876	0.05398093	-2.60756452	0.01979853	Negative
ENSBTAG00000007544	-0.13651384	0.05426754	-2.51557098	0.02376359	Negative
ENSBTAG00000005133	-0.13457171	0.0541434	-2.48546831	0.02521838	Negative
ENSBTAG00000019734	-0.12569642	0.03847322	-3.26711432	0.00519777	Negative
ENSBTAG00000013501	-0.12422353	0.04560437	-2.72393895	0.01568679	Negative
ENSBTAG00000046010	-0.1220878	0.02664981	-4.5811878	0.00036018	Negative
ENSBTAG00000007312	-0.11141306	0.0432807	-2.57419707	0.02115722	Negative
ENSBTAG00000031146	-0.11033046	0.05067837	-2.1770722	0.04585583	Negative
ENSBTAG00000017890	-0.10743622	0.03731086	-2.87948917	0.0114614	Negative
ENSBTAG00000013203	-0.1042827	0.02875495	-3.62659996	0.00248629	Negative
ENSBTAG00000005940	-0.10376251	0.0389301	-2.66535419	0.01764141	Negative
ENSBTAG00000014005	-0.09411462	0.02882059	-3.26553396	0.00521463	Negative
ENSBTAG00000010590	-0.08704432	0.03122643	-2.78752109	0.01380279	Negative
ENSBTAG00000038	-	0.04063893	-	0.049639	Negative

178	0.0867755		2.13527992	19	
ENSBTAG00000013413	-0.08127314	0.02703282	-3.00646143	0.00885544	Negative
ENSBTAG00000013666	-0.08040193	0.02256593	-3.56297794	0.0028328	Negative
ENSBTAG00000010709	-0.07892554	0.02596053	-3.0402133	0.00826683	Negative
ENSBTAG00000019354	-0.07544997	0.02456171	-3.07185325	0.00775008	Negative
ENSBTAG00000021321	-0.07017458	0.02401558	-2.9220447	0.01051376	Negative
ENSBTAG00000020539	-0.06616399	0.02602684	-2.54214445	0.02254626	Negative
ENSBTAG00000006016	-0.0639426	0.01941223	-3.2939347	0.00491971	Negative
ENSBTAG00000007678	-0.06183116	0.02055426	-3.00819189	0.00882429	Negative
ENSBTAG00000015764	-0.06058901	0.02300914	-2.63325806	0.01880984	Negative
ENSBTAG00000020643	-0.05588442	0.01790107	-3.1218473	0.00699777	Negative
ENSBTAG00000018834	-0.05187079	0.02260771	-2.29438494	0.03661943	Negative
ENSBTAG00000018920	-0.05006294	0.01840209	-2.7205036	0.01579538	Negative
ENSBTAG00000030749	-0.0485822	0.01268125	-3.8310322	0.0016361	Negative

	8		1		
ENSBTAG00000009617	-0.04751548	0.02044159	-2.32445136	0.03454984	Negative
ENSBTAG00000004850	-0.03932963	0.01388678	-2.83216383	0.01261329	Negative
ENSBTAG00000022227	-0.03915957	0.01236349	-3.16735577	0.00637593	Negative
ENSBTAG00000001440	-0.03496722	0.01308734	-2.67183515	0.01741412	Negative
ENSBTAG00000036183	-0.03361861	0.00945484	-3.5557053	0.00287538	Negative
ENSBTAG00000013211	-0.03358391	0.01133875	-2.96187048	0.0096966	Negative
ENSBTAG00000001361	-0.03038204	0.01355355	-2.24162995	0.04053418	Negative
ENSBTAG00000011482	-0.02831159	0.00850547	-3.3286344	0.0045818	Negative
ENSBTAG00000021134	-0.02473133	0.01123073	-2.20211226	0.04371896	Negative
ENSBTAG00000003062	-0.02449594	0.00672886	-3.640431	0.00241678	Negative
ENSBTAG00000004489	-0.02395662	0.00707308	-3.38701637	0.00406464	Negative
ENSBTAG00000009446	-0.02293421	0.00701945	-3.26723882	0.00519644	Negative
ENSBTAG00000018841	-0.0187683	0.00825029	-2.274872	0.03802423	Negative

	6				
ENSBTAG00000012252	-0.01859476	0.00569077	-3.2675284	0.00519336	Negative
ENSBTAG00000004732	-0.01847034	0.00616229	-2.99731648	0.0090219	Negative
ENSBTAG00000004495	-0.01485962	0.0063191	-2.35154214	0.03277975	Negative
ENSBTAG00000033891	-0.01420424	0.00619977	-2.29109235	0.03685301	Negative
ENSBTAG00000021617	-0.01320909	0.00496415	-2.660897	0.01779938	Negative
ENSBTAG00000000315	-0.01184373	0.00466401	-2.53938522	0.02266983	Negative
ENSBTAG00000002651	-0.01126448	0.00440992	-2.55435188	0.02200724	Negative
ENSBTAG00000020806	-0.01113614	0.00376611	-2.9569392	0.0097943	Negative
ENSBTAG00000002487	-0.01015052	0.00255391	-3.97450723	0.00122104	Negative
ENSBTAG00000045742	-0.00948609	0.00443327	-2.13975089	0.04922107	Negative
ENSBTAG00000018523	-0.00943569	0.00417517	-2.2599507	0.03913233	Negative
ENSBTAG00000022520	-0.0089411	0.00246523	-3.62688872	0.00248482	Negative
ENSBTAG00000002726	-0.0088644	0.00341336	-2.5969981	0.02021945	Negative

	8		7		
ENSBTAG00000012365	-0.008664	0.00319788	-2.70929686	0.01615471	Negative
ENSBTAG00000014105	-0.00826226	0.00195572	-4.22467254	0.00073535	Negative
ENSBTAG00000007943	-0.00788804	0.00306527	-2.57336054	0.02119241	Negative
ENSBTAG00000010322	-0.00735949	0.00234939	-3.13251119	0.00684689	Negative
ENSBTAG00000021013	-0.00708734	0.00246622	-2.87376266	0.01159513	Negative
ENSBTAG00000004190	-0.00692056	0.00242359	-2.85550346	0.01203177	Negative
ENSBTAG00000018417	-0.00665636	0.00277911	-2.39513937	0.03010955	Negative
ENSBTAG00000016199	-0.00616373	0.0018795	-3.27944405	0.00506805	Negative
ENSBTAG00000012989	-0.00550724	0.00236537	-2.32827723	0.03429452	Negative
ENSBTAG00000019020	-0.00489767	0.00171381	-2.85776813	0.01197675	Negative
ENSBTAG00000002178	-0.00424802	0.00199059	-2.13405342	0.04975446	Negative
ENSBTAG00000038532	-0.00398678	0.00177564	-2.24527058	0.04025193	Negative
ENSBTAG00000016708	-0.0024601	0.00091125	-2.6997313	0.01646765	Negative

	3		8		
ENSBTAG00000005970	-0.0019503	0.00080146	-2.43343623	0.02793499	Negative
ENSBTAG00000019225	-0.00045956	0.00018911	-2.43007541	0.0281197	Negative
ENSBTAG00000002255	-0.00033984	0.00011263	-3.01718615	0.00866408	Negative
ENSBTAG00000003362	-0.00020538	9.516E-05	-2.15826489	0.0475241	Negative
ENSBTAG00000017294	-4.1755E-05	1.9553E-05	-2.13545767	0.0496225	Negative
ENSBTAG00000045376	-0.80225676	0.43916193	-1.82679031	0.08769651	NA
ENSBTAG00000043350	-0.46289896	0.22942329	-2.01766338	0.06188425	NA
ENSBTAG00000023986	-0.45674487	0.24792414	-1.84227669	0.08529009	NA
ENSBTAG00000003638	-0.22527317	0.13284549	-1.69575324	0.11058282	NA
ENSBTAG00000015644	-0.16185694	0.08507608	-1.90249652	0.07648276	NA
ENSBTAG00000016095	-0.13003716	0.08024298	-1.62054243	0.12594322	NA
ENSBTAG00000017622	-0.12155451	0.06484456	-1.87455224	0.08046305	NA
ENSBTAG00000038055	-0.1051278	0.06300047	-1.6686841	0.1159131	NA

	9		5		
ENSBTAG00000009782	-0.07783522	0.04314302	-1.8041211	0.09132784	NA
ENSBTAG00000002121	-0.05409118	0.02566119	-2.10789808	0.05227221	NA
ENSBTAG00000031029	-0.05044209	0.02567113	-1.96493426	0.06822196	NA
ENSBTAG00000031792	-0.04994504	0.02820818	-1.77058741	0.09694363	NA
ENSBTAG00000021007	-0.04688778	0.02242788	-2.09060212	0.05400105	NA
ENSBTAG00000001675	-0.04141989	0.02018197	-2.05232136	0.05801576	NA
ENSBTAG00000008756	-0.03955057	0.03072258	-1.2873454	0.21748377	NA
ENSBTAG00000030301	-0.03929078	0.024148	-1.62708181	0.12453839	NA
ENSBTAG00000006730	-0.03400945	0.01683857	-2.01973466	0.0616466	NA
ENSBTAG00000001997	-0.02655782	0.01606945	-1.65269029	0.11916598	NA
ENSBTAG00000012002	-0.02440408	0.01445996	-1.68769995	0.11214591	NA
ENSBTAG00000034985	-0.02222335	0.01575208	-1.4108196	0.17870009	NA
ENSBTAG00000020780	-0.0198288	0.0119573	-1.6583078	0.11801458	NA

	8		9		
ENSBTAG00000021177	-0.01400239	0.00845347	-1.6564074	0.11840303	NA
ENSBTAG00000032092	-0.01141931	0.00569751	-2.00426228	0.0634421	NA
ENSBTAG00000005182	-0.01138764	0.0055606	-2.04791587	0.05849492	NA
ENSBTAG00000020368	-0.01013273	0.1030949	-0.09828544	0.92300702	NA
ENSBTAG00000007402	-0.0084496	0.00820953	-1.02924301	0.31967705	NA
ENSBTAG00000009677	-0.00742374	0.00381109	-1.94792974	0.07038791	NA
ENSBTAG00000019448	-0.00591115	0.00305826	-1.93284752	0.0723608	NA
ENSBTAG00000000250	-0.00581799	0.0035809	-1.62473067	0.12504192	NA
ENSBTAG00000038748	-0.00354399	0.0075855	-0.46720625	0.64706701	NA
ENSBTAG00000019887	-0.00334374	0.00164867	-2.02813915	0.06069083	NA
ENSBTAG00000003492	-0.003243	0.00219078	-1.48029643	0.15949074	NA
ENSBTAG00000007952	-0.00265752	0.00151623	-1.75271223	0.10005975	NA
ENSBTAG00000002333	-0.0023705	0.00126242	-1.8777774	0.07999438	NA

	5		7		
ENSBTAG00000021523	-0.0013257	0.00067191	-1.97303416	0.0672116	NA
ENSBTAG00000003196	-0.0012215	0.00057951	-2.10783257	0.05227866	NA
ENSBTAG00000017143	-0.00068943	0.00037237	-1.85145797	0.08389134	NA
ENSBTAG00000007554	-0.00057607	0.00032623	-1.7658602	0.09775928	NA
ENSBTAG00000009846	0.0015092	0.00543968	0.27744192	0.785228	NA
ENSBTAG00000004322	0.00197917	0.00132466	1.49409885	0.15588666	NA
ENSBTAG00000004037	0.00335877	0.0025699	1.30696768	0.21090599	NA
ENSBTAG00000015188	0.00394441	0.0019128	2.06210773	0.05696418	NA
ENSBTAG00000017863	0.00452699	0.00215826	2.09751252	0.0533041	NA
ENSBTAG00000013118	0.00519651	0.00371511	1.39875022	0.18222355	NA
ENSBTAG00000000706	0.00626667	0.0036336	1.72464606	0.10512999	NA
ENSBTAG00000001785	0.00916861	0.00839562	1.09207055	0.29203252	NA
ENSBTAG00000010069	0.00920032	0.00727429	1.26477367	0.22525018	NA
ENSBTAG00000025257	0.01069889	0.00544326	1.96552899	0.06814731	NA
ENSBTAG00000008182	0.01088411	0.00713906	1.52458563	0.14816698	NA
ENSBTAG00000031430	0.01148708	0.00578639	1.98519006	0.06572076	NA
ENSBTAG00000000507	0.01169633	0.00692132	1.68989983	0.11171703	NA

ENSBTAG00000010349	0.01336373	0.00871611	1.53322168	0.14603956	NA
ENSBTAG00000013371	0.01349142	0.01079085	1.25026421	0.23035677	NA
ENSBTAG00000025250	0.01504279	0.00847807	1.77431768	0.09630422	NA
ENSBTAG00000014127	0.01540345	0.0121565	1.26709593	0.22444119	NA
ENSBTAG00000002362	0.0156327	0.0105661	1.47951441	0.15969701	NA
ENSBTAG00000004556	0.02204114	0.01422117	1.54988178	0.14200824	NA
ENSBTAG00000013802	0.02244115	0.01196375	1.87576287	0.08028684	NA
ENSBTAG00000019716	0.02375018	0.01805501	1.31543406	0.20811729	NA
ENSBTAG00000006240	0.0281645	0.01774605	1.58708517	0.13334515	NA
ENSBTAG00000014669	0.03137162	0.01472429	2.13060312	0.05008006	NA
ENSBTAG00000005431	0.03222328	0.0199071	1.61868281	0.12634519	NA
ENSBTAG00000023776	0.03475639	0.01975582	1.75929894	0.09890141	NA
ENSBTAG00000037811	0.04123814	0.02415709	1.7070825	0.108416	NA
ENSBTAG00000020355	0.04710786	0.03360445	1.40183372	0.18131801	NA
ENSBTAG00000046409	0.04820446	0.03058968	1.57584079	0.13591476	NA
ENSBTAG00000006256	0.05696005	0.03677907	1.54870847	0.14228904	NA
ENSBTAG00000034885	0.05936647	0.04088589	1.45200377	0.16709572	NA
ENSBTAG00000020872	0.06179896	0.04173865	1.48061713	0.15940622	NA
ENSBTAG00000013443	0.06603594	0.06356937	1.03880111	0.31535336	NA
ENSBTAG00000004	0.0738241	0.03886741	1.8993846	0.076917	NA

894	6		8	08	
ENSBTAG00000006685	0.07756225	0.04654777	1.66629351	0.11639437	NA
ENSBTAG00000003880	0.08315881	0.04518135	1.84055616	0.08555451	NA
ENSBTAG00000007101	0.08454359	0.04238605	1.99460856	0.06458634	NA
ENSBTAG00000006894	0.10000547	0.05095047	1.96279782	0.06849074	NA
ENSBTAG00000026753	0.10548281	0.06253237	1.68685127	0.11231175	NA
ENSBTAG00000011487	0.11363693	0.05895389	1.92755628	0.07306468	NA
ENSBTAG00000015821	0.13956029	0.07745853	1.801742	0.09171656	NA
ENSBTAG00000038042	0.15774759	0.08139218	1.93811744	0.07166585	NA
ENSBTAG00000014113	0.19218173	0.10368935	1.85343747	0.08359246	NA
ENSBTAG00000037735	0.21880333	0.13029758	1.67925852	0.1138049	NA
ENSBTAG00000048112	0.24902514	0.16066542	1.54996104	0.14198929	NA
ENSBTAG00000044275	0.26345012	0.13244871	1.98907279	0.06525093	NA
ENSBTAG00000011551	0.36067833	0.18717884	1.9269183	0.07314996	NA
